# MYC–SNHG7–miR-34a-5p Feedback Loop Orchestrates GSDMD-Dependent Pyroptosis in Hepatocellular Carcinoma

**DOI:** 10.3390/ijms27198441

**Published:** 2026-09-22

**Authors:** Houemakou Rimaud Djidonou, Laís de Carvalho Gonçalves, Djorkaeff O. Fontinele, Daner A. Silveira, Marialva Sinigaglia, Joao Paulo M. S. Lima, José Carlos M. Mombach, Shantanu Gupta

**Affiliations:** 1 Bioinformatics Multidisciplinary Environment-BioME—Digital Metropole Institute, Federal University of Rio Grande do Norte, Natal 59076-550, RN, Brazil; houemakou.rimaud.djidonou.866@ufrn.edu.br (H.R.D.); lais.goncalves.034@ufrn.edu.br (L.d.C.G.); djorkaeffof@gmail.com (D.O.F.); jpmslima@imd.ufrn.br (J.P.M.S.L.); 2 Children’s Cancer Institute, Porto Alegre 90620-110, RS, Brazil; d.silveira.bioinfo@ici.ong (D.A.S.); msinigaglia@ici.ong (M.S.); 3 Department of Biochemistry, Federal University of Rio Grande do Norte, Natal 59076-550, RN, Brazil; 4 Departamento de Física, Universidade Federal de Santa Maria, Santa Maria 97105-900, RS, Brazil; jcmombach@ufsm.br

**Keywords:** hepatocellular carcinoma, pyroptosis, GSDMD, SNHG7, miR-34a-5p, MYC, Boolean network modeling, drug resistance

## Abstract

Pyroptosis is an immunogenic form of regulated cell death mediated by NLRP3 inflammasome activation, caspase-1, and gasdermin D. Unlike apoptosis, pyroptosis promotes anti-tumor immunity through IL-1β and IL-18 release, making it a promising therapeutic target in hepatocellular carcinoma (HCC). Although the SNHG7/miR-34a/SIRT1 axis has been experimentally linked to NLRP3-dependent pyroptosis, whether it functions as a systems-level regulatory module coordinating the balance between drug resistance and multiple cell-fate outcomes remains unknown. To address this question, we developed a network medicine framework using a Boolean network model integrating drug resistance, senescence, apoptosis, and pyroptosis pathways in HCC. The model was constructed from experimentally validated molecular interactions and independently validated using gain- and loss-of-function perturbation studies that were not used during model construction, demonstrating strong agreement with experimental observations. Our analysis identified a previously unreported MYC–SNHG7–miR-34a double-negative feedback loop, establishing miR-34a as a central regulatory hub controlling cell-fate decisions. Perturbation analysis predicted that SNHG7 inhibition shifts cells from drug resistance toward pyroptosis, identifying restoration of miR-34a activity as a potential strategy to induce immunogenic cell death. These findings provide a systems-level framework for therapeutic targeting of the MYC–SNHG7–miR-34a axis in HCC.

## 1. Introduction

Liver cancer remains one of the most lethal malignancies worldwide, with hepatocellular carcinoma (HCC) accounting for the majority of cases and exhibiting high rates of therapeutic resistance and poor prognosis [1]. Treatment failure is largely driven by the ability of tumor cells to evade multiple forms of programmed cell death [2]. Among these, pyroptosis—a lytic, inflammatory form of cell death executed by gasdermin D (GSDMD) following NLRP3 inflammasome (NLRP3)–mediated Caspase-1 activation, has emerged as a critical tumor-suppressive mechanism [3,4]. Unlike apoptosis, pyroptosis promotes anti-tumor immunity through the release of Interleukin-1 beta (IL-1β) and Interleukin-18 (IL-18) [4]. However, cancer cells frequently suppress pyroptotic signaling, contributing to therapy resistance and immune evasion [5].

Non-coding RNAs (ncRNAs), particularly microRNAs (miRNAs) and long non-coding RNAs (lncRNAs), are key regulators of cell-death pathways in cancer [6]. miR-34a-5p (hereafter miR-34a) is a well-characterized tumor suppressor that represses multiple oncogenes, including MYC proto-oncogene (MYC) and Sirtuin 1 (SIRT1) [7,8]. Its expression is frequently downregulated in HCC, correlating with poor prognosis and treatment failure [9]. The lncRNA Small nucleolar RNA host gene 7 (hereafter SNHG7) promotes tumor progression and chemoresistance by functioning as a competing endogenous RNA (ceRNA) for miR-34a [10]. Recent experimental evidence has demonstrated that the SNHG7/miR-34a/SIRT1 axis regulates NLRP3-dependent pyroptosis in HCC cells [11]. However, the systems-level logic underlying the balance between drug resistance, senescence, apoptosis, and pyroptosis remains unclear.

Boolean network modeling is a widely used systems biology approach for studying complex gene regulatory networks (GRNs) in cancer [12,13,14]. In this representation, GRNs are modeled as directed graphs with nodes representing molecular components and edges representing activating or inhibitory interactions [14]. Unlike quantitative approaches that require detailed kinetic parameters, Boolean networks provide a qualitative but robust description, making them suitable when experimental data are limited [13,14]. Each component is assigned a binary state (ON/OFF), and its dynamics are governed by logical rules derived from experimentally validated interactions. The long-term behavior of the system is characterized by attractors, including stable states and cyclic attractors, which represent distinct cellular phenotypes and cell-fate decisions. Regulatory circuits, particularly positive and negative feedback loops, strongly influence these dynamics and attractor stability [15,16]. Overall, Boolean network modeling is a powerful systems biology approach for elucidating cancer progression, therapeutic response, and cell-fate regulation [17,18,19,20,21]. Further details are provided in the Methods.

While several targeted therapies, including sorafenib, lenvatinib, and regorafenib, have been approved for advanced HCC, their efficacy is often limited by acquired resistance [22,23,24]. Notably, these agents have been shown to induce DNA damage and modulate the DNA damage response (DDR) as part of their mechanism of action [25,26,27]. Furthermore, emerging combination strategies, such as PARP inhibitors with radiotherapy or immunotherapy, are being actively investigated in HCC [28,29,30]. Therefore, understanding the regulatory logic governing cell-fate decisions under DDR conditions remains clinically relevant for identifying novel therapeutic vulnerabilities in HCC.

In this study, we construct a Boolean network model of the MYC/SNHG7/miR-34a/SIRT1 regulatory network in HCC to investigate its role in governing drug resistance, senescence, apoptosis, and pyroptosis-associated cell-fate decisions. Through attractor analysis, perturbation simulations, and feedback-loop analysis, we identify a previously unrecognized MYC/SNHG7/miR-34a regulatory loop that functions as a multistable switch between drug resistance and cell-death programs.

## 2. Results

### 2.1. Network Architecture and Wild-Type Attractor Analysis

The SNHG7/miR-34a/SIRT1 axis has recently emerged as an important regulator of HCC progression and NLRP3-dependent pyroptosis. However, how this regulatory axis integrates with DNA damage signaling and other cell-fate pathways to govern drug resistance, senescence, apoptosis, and pyroptosis remains poorly understood. To address this question, we constructed a Boolean network model describing the molecular interactions linking the DNA damage response (DDR), the SNHG7/miR-34a/SIRT1 axis, and downstream cell-fate decisions in HCC. The model consists of 32 nodes representing proteins, miRNAs, lncRNAs, and cellular phenotypes connected through 76 regulatory interactions (Figure 1A). In the network representation, miR-34a is depicted as a yellow oval node. In contrast, SNHG7 is represented as a pink rectangular node. Proteins and transcription factors are shown as white nodes. Black arrows and hammerhead lines denote activating and inhibitory interactions, respectively, while red hammerhead lines indicate miRNA-mediated repression of target genes. The logical rules governing node activity are provided in Table S1, which also includes the full names of the network components, their biological descriptions, and the supporting literature.

There is one input in our model, namely DDR (Figure 1A). Upon DDR, DDR signaling activates Ataxia telangiectasia mutated (ATM) [31] and AMP-activated protein kinase (AMPK) [32], which subsequently promote Tumor protein p53 (p53) activation [33,34]. Activated p53 induces the expression of several downstream targets, including Cyclin-dependent kinase inhibitor 1A (p21) [35], p53-inducible nuclear protein 1 (p53INP1) [36], and miR-34a [37], thereby initiating tumor-suppressive programs. Similar to previous studies describing p53 dynamics during the DDR [36], our model distinguishes between two functional states of p53. p53_A represents p53 phosphorylated at Ser15 and Ser20 and is primarily associated with growth arrest and senescence [38], whereas p53_K represents p53 phosphorylated at Ser46 and promotes apoptotic [39] and pyroptotic responses [40]. The transition between these two functional states is regulated by the opposing activities of Wild-type p53-induced phosphatase 1 (Wip1) [41] and p53INP1 [36]. p53_A induces p21 expression [35], leading to cell-cycle arrest and senescence, whereas p53_K activates pro-death regulators, including p53-upregulated modulator of apoptosis (PUMA) [39], BCL2-associated X protein (BAX) [42], and caspase signaling, thereby promoting apoptotic cell death.

miR-34a functions as a key tumor suppressor downstream of p53 and negatively regulates several oncogenic targets, including MYC [43], SIRT1 [44], E2F transcription factor 1 (E2F1) [45], and B-cell lymphoma 2 (BCL2) [9]. MYC positively regulates the expression of the SNHG7 [46], whereas SNHG7 functions as a ceRNA that suppresses miR-34a activity [47]. Consequently, MYC, SNHG7, and miR-34a establish a regulatory feedback loop that controls the balance between oncogenic and tumor-suppressive signaling.

Recent experimental evidence demonstrated that SNHG7 suppresses NLRP3-dependent pyroptosis through regulation of the miR-34a/SIRT1 axis in HCC cells [11]. Consistent with these observations, SIRT1 negatively regulates NLRP3 activity in our model [48]. Activation of NLRP3 promotes Caspase-1 activation [49], which subsequently activates GSDMD and induces pyroptotic cell death. Thus, the SNHG7/miR-34a/SIRT1 axis provides a mechanistic link between oncogenic signaling and inflammasome-mediated pyroptosis. Furthermore, p53_K contributes to pyroptotic signaling through activation of pro-death pathways [50], whereas suppression of SIRT1 enhances NLRP3 activation and favors pyroptotic commitment.

In parallel, MYC, E2F1, AKT serine/threonine kinase (AKT) [51,52], mouse double minute 2 homolog (MDM2) [53], and Cyclin-dependent kinase 4/6-Cyclin D complex (CDK4/6-Cyclin D) [54] signaling promote proliferation and resistance-associated phenotypes. Retinoblastoma protein 1 (RB1) negatively regulates cell-cycle progression through inhibition of E2F1 activity [55], whereas p21 suppresses CDK4/6-Cyclin D [56] activity and reinforces growth arrest. Through these interconnected regulatory pathways, we present this Boolean network model of HCC cell-fate regulation based on the above experimentally validated interactions (Figure 1A). The model provides a systems-level framework for investigating how the MYC–SNHG7–miR-34a regulatory axis controls drug resistance and pyroptosis-associated cell-fate decisions in HCC.

We next examined the intrinsic dynamics of the network under wild-type conditions to determine the stable cellular states supported by the model. Our analysis revealed five distinct stable attractors (Figure 1B). In the absence of DNA damage (DDR OFF), the system converges to a single attractor corresponding to Proliferation, characterized by activation of MDM2, AKT, CDK4/6, Cyclin D, E2F1, SIRT1, and SNHG7. Under DDR ON conditions, four additional attractors emerge, which are described in detail below.

Under DDR ON, the system exhibited four attractors. The drug resistance state is defined here as a generic survival phenotype—a stable attractor state reflecting the ability of cancer cells to survive therapeutic challenge, rather than resistance to any specific drug. The drug resistance state is defined by sustained MYC, E2F1, Wip1, SNHG7, and SIRT1 activity. The senescence state is characterized by p53_A, p21, and miR-34a activation. The pyroptotic state shows activity of p53_K, NLRP3, caspase-1, and GSDMD, independent of Caspase-3 and Caspase-9. The apoptosis state is marked by p53_K, Caspase-3, and Caspase-9 activity, with GSDMD absent.

In Figure 1B, these five attractors are represented as columns from left to right: Proliferation (column 1, DDR OFF), Drug Resistance (column 2, DDR ON), Senescence (column 3, DDR ON), Pyroptosis (column 4, DDR ON), and Apoptosis (column 5, DDR ON).

Monte Carlo simulations (100,000 runs) quantified the probability of each attractor under two conditions. When the DDR input was left free (neither fixed ON nor OFF), the system converged predominantly to survival-associated states: Proliferation (50%), Drug Resistance (47%), Senescence (1%), Pyroptosis (1%), and Apoptosis (1%) (see Figure 1C). When DDR was fixed ON, Proliferation was completely abolished (0%), while Drug Resistance remained the dominant attractor (93%), followed by Senescence (3%), Pyroptosis (2%), and Apoptosis (2%) (Figure 1D).

### 2.2. Model Validation with Known Experimental Observations in HCC

To assess predictive performance, the model was validated against independent experimental studies not used in network construction [11,57,58,59]. In silico gain- and loss-of-function perturbations of key regulatory nodes, including SNHG7 gain-of-function, miR-34a gain- and loss-of-function, and SIRT1 gain-of-function, were performed under DNA damage conditions (Input = ON) (Figure 2), consistent with the present model’s DNA damage response framework. The resulting phenotypic predictions were then compared with the corresponding experimental observations reported in the literature.

SNHG7 overexpression locked the system into the drug resistance attractor, characterized by activation of SIRT1 [11], while pyroptosis, apoptosis, and senescence remained inactive. This is consistent with the oncogenic role of SNHG7 and its ability to inhibit miR-34a and promote survival in HCC [11].

miR-34a overexpression produced three distinct attractors depending on the downstream signaling context. The senescence attractor was marked by activation of p53, p53_A, p21, and miR-34a, aligning with the role of miR-34a in cell-cycle arrest through targeting CDK4/6 and Cyclin D [57] and in telomerase inhibition through targeting MYC [58]. The pyroptosis attractor was defined by activation of p53, p53_K, miR-34a, NLRP3, Caspase-1, and GSDMD, recapitulating the experimental observation that miR-34a upregulation enhances NLRP3-dependent pyroptosis in HCC cells [11]. The apoptosis attractor was characterized by activation of miR-34a, Caspase-3, and Caspase-9, consistent with the known pro-apoptotic function of miR-34a through SIRT1 [57] and BCL2 suppression and caspase activation in HCC cells [59].

Conversely, miR-34a knockout [11,57,59] and SIRT1 overexpression [11,57] both stabilized the drug resistance attractor, characterized by activation of SIRT1, BCL2, and CDK4/6-CyclinD, confirming that miR-34a loss or SIRT1 activation is sufficient to block pyroptotic and apoptotic commitment.

Collectively, these perturbation analyses demonstrate that the model faithfully recapitulates independent experimental observations and establishes the SNHG7/miR-34a/SIRT1 axis as a central determinant of cell fate in HCC.

### 2.3. Feedback Loop Analysis

Feedback loops (also known as circuits) are critical in regulating cell fate, with positive feedback loops stabilizing phenotypic states and negative loops often contributing to oscillations. We identified several regulatory circuits within the network, focusing on those composed of no more than three components. Many of these circuits have been experimentally validated (Table S2).

Among these, one circuit, the MYC/SNHG7/miR-34a double-negative loop, has not been previously reported. Its individual interactions are supported by published data (Table 1), but their assembly into a closed loop is novel.

To analyse the role of this circuit, we performed two perturbation scenarios under DDR (input) ON conditions (see Figure 3): (A) MYC and SNHG7 overexpression (E1) combined with miR-34a knockdown (KO), and (B) miR-34a overexpression (E1) combined with MYC and SNHG7 knockdown (KO). Monte Carlo simulations (100,000 runs) were used to quantify the phenotypic distributions.

In the first scenario, miR-34a knockdown (KO) with MYC and SNHG7 (E1) overexpression locked the system into a pure drug-resistant state (Figure 3A). In contrast, miR-34a overexpression (E1) with MYC and SNHG7 knockdown (KO) eliminated drug resistance and shifted the system toward cell death, producing 35% senescence, 15% apoptosis, and 50% pyroptosis (Figure 3B). These results suggest that the MYC/SNHG7/miR-34a loop functions as a multistable switch, with miR-34a activation sufficient to overcome drug resistance and drive cell death.

## 3. Discussion

In this study, we developed a Boolean network model of the SNHG7/miR-34a/SIRT1 axis to investigate cell-fate decisions in HCC (Figure 1A). Wild-type simulations identified five stable attractors: proliferation, drug resistance, senescence, pyroptosis, and apoptosis, each defined by distinct activation patterns of key regulators. In the absence of DDR, the proliferation attractor was marked by activation of MDM2, AKT, and CDK4/6. Under DDR-ON conditions, four additional attractors emerged: the drug resistance attractor, characterized by sustained MYC, E2F1, Wip1, SNHG7, and SIRT1; the senescence attractor, defined by p53_A, p21, and miR-34a; the pyroptosis attractor, marked by p53_K, NLRP3, caspase-1, and GSDMD; and the apoptosis attractor, characterized by p53_K, caspase-3, and caspase-9 (Figure 1B).

Conventional pathway analysis typically represents signaling as linear cascades, which can obscure emergent properties such as feedback loops and context-dependent outcomes. Boolean modeling overcomes this limitation by representing regulatory interactions as logical rules, enabling systematic investigation of how the same input can produce distinct cell-fate outcomes depending on network state—a critical feature for studying complex decisions where multiple signals converge on shared regulators. In this study, this approach allowed us to identify that the MYC–SNHG7–miR-34a axis operates as a multistable switch, a property that would be difficult to infer from linear pathway models alone.

To assess whether our model recapitulates known experimental outcomes, we systematically perturbed key regulatory nodes via gain- and loss-of-function simulations (See Figure 2). SNHG7 overexpression locked the system into drug resistance, consistent with its oncogenic role and ability to inhibit miR-34a [11]. miR-34a overexpression produced three distinct outcomes: senescence, pyroptosis, and apoptosis, aligning with its known functions in cell-cycle arrest by targeting CDK4/6 and Cyclin D [57], and senescence by targeting MYC [58], NLRP3-dependent pyroptosis by targeting SIRT1 [11], and caspase-mediated apoptosis [59]. Conversely, miR-34a knockout and SIRT1 overexpression both stabilized the drug-resistant state, confirming that loss of miR-34a or activation of SIRT1 is sufficient to block pyroptotic and apoptotic commitment [11,57]. Collectively, these perturbations confirm that the model faithfully recapitulates independent experimental observations and establishes the SNHG7/miR-34a/SIRT1 axis as a central determinant of cell fate in HCC.

A central finding of this study is the identification of a previously unrecognized MYC/SNHG7/miR-34a regulatory circuit. Our network analysis revealed that MYC activates SNHG7, SNHG7 suppresses miR-34a through competitive sponging, and miR-34a represses MYC, forming a double-negative feedback architecture. Monte Carlo simulations demonstrate that this circuit functions as a multistable switch. When MYC and SNHG7 are overexpressed together with miR-34a knockdown, the system locks into a pure drug-resistant state. In contrast, when MYC and SNHG7 are knocked down and miR-34a is overexpressed, the system shifts toward cell death, producing senescence, apoptosis, and pyroptosis (Figure 3). These findings suggest that the MYC/SNHG7/miR-34a loop contributes to context-dependent cell-fate decisions, with miR-34a acting as a key determinant of the transition between resistant and death-associated states.

The biological relevance of this circuit extends beyond its architectural novelty. By functioning as a multistable switch, the MYC–SNHG7–miR-34a loop enables HCC cells to toggle between distinct functional states: a miR-34a-high/p53-active state associated with drug sensitivity and cell death (including pyroptosis and apoptosis), and a MYC/SNHG7-dominant state characterized by sustained proliferation and therapy resistance. Importantly, our model predicts that miR-34a restoration not only induces apoptosis but also promotes pyroptosis through the SIRT1/NLRP3/GSDMD axis, positioning pyroptosis as a key immunogenic outcome of disrupting the oncogenic circuit. This switch-like behavior provides a mechanistic basis for phenotypic plasticity, allowing cancer cells to adapt to therapeutic pressure by shifting the population toward the resistant state, a process that contributes to tumor heterogeneity and treatment failure. Importantly, the circuit integrates oncogenic signals (MYC), non-coding RNA regulation (SNHG7/miR-34a), and stress-responsive pathways (p53/SIRT1), positioning it as a central hub that coordinates cellular adaptation to genotoxic stress. From a therapeutic standpoint, disrupting this circuit—for example through miR-34a restoration or SNHG7 inhibition—may reverse the resistant phenotype and re-sensitize HCC cells to therapy, as suggested by our perturbation analyses.

Building on these observations, we suggest that the SNHG7-miR-34a axis is embedded within a larger regulatory module capable of stabilizing distinct cellular outcomes (Figure 4). Previous experimental studies have independently demonstrated that miR-34a regulates apoptosis through BCL2 [59], senescence via MYC pathway [58], and GSDMD-dependent pyroptosis through the SNHG7/miR-34a/SIRT1 axis [11]. By integrating these experimentally validated pathways into a unified systems-level framework, our analysis reveals how the upstream regulator miR-34a coordinates alternative cell-fate decisions. From a network medicine perspective, the MYC–SNHG7–miR-34a circuit therefore functions as a regulatory module whose perturbation rewires the balance between drug resistance, senescence, apoptosis, and pyroptosis rather than affecting a single signaling pathway.

Notably, senescence [58] and apoptosis [57,59] were retained in the model because they represent well-established downstream outcomes of miR-34a and p53 signaling. Their inclusion was necessary to capture the broader spectrum of competing cell-fate decisions rather than treating pyroptosis as an isolated endpoint. Within this framework, pyroptosis emerges as the most distinctive outcome associated with disruption of the MYC/SNHG7/SIRT1 oncogenic program, highlighting a potential link between non-coding RNA regulation and inflammatory cell death.

From a therapeutic perspective, our simulations suggest that miR-34a may function as a systems-level regulator capable of simultaneously suppressing multiple resistance-associated pathways, including MYC, SNHG7, SIRT1, BCL2, and E2F1. Rather than targeting a single oncogenic effector, restoration of miR-34a activity is predicted to rewire the broader regulatory network controlling therapy response. The persistence of senescence under certain perturbation conditions further suggests that combination strategies may be required to achieve complete elimination of resistant tumor cells. This is consistent with the emerging view that senescence can, in some contexts, represent a therapy-refractory state that actively contributes to drug resistance and tumor relapse [61].

Several strategies are currently being explored to restore miR-34a activity in cancer, including synthetic miRNA mimics, viral vector-based delivery, and nanoparticle formulations. The miR-34a mimic MRX34 was the first miRNA-based therapeutic to enter clinical trials, demonstrating the feasibility of miRNA replacement therapy in cancer patients, although hepatotoxicity limited its further development [62]. In HCC, targeted delivery approaches, such as lipid nanoparticles decorated with ligands for the asialoglycoprotein receptor (ASGPR) on hepatocytes, are being investigated to improve tumor specificity and reduce off-target effects [63,64]. Additionally, small-molecule inducers of endogenous miR-34a expression represent an alternative strategy that may bypass some of the delivery challenges associated with synthetic mimics [65]. While these approaches hold promise, their clinical translation requires careful optimization of delivery systems, patient stratification, and combination with standard therapies to maximize therapeutic benefit. For HCC specifically, the presence of the MYC–SNHG7–miR-34a feedback loop provides a strong mechanistic rationale for miR-34a restoration, as it would simultaneously disrupt oncogenic MYC signaling and relieve SNHG7-mediated suppression of pyroptosis, offering a dual therapeutic benefit.

Several limitations should be acknowledged. First, Boolean modeling simplifies molecular activity into binary states and does not capture quantitative kinetics or post-translational regulation. Future work could extend this Boolean framework by incorporating quantitative or multivalued modeling approaches. Ordinary differential equation (ODE) models could capture graded expression dynamics and dose-dependent responses, such as the relationship between miR-34a levels and apoptosis induction. Multivalued logical models could distinguish intermediate activity states, which may be relevant for understanding partial pathway activation in heterogeneous tumor populations. Agent-based or spatial models could further incorporate tumor microenvironmental factors, such as hypoxia or immune cell infiltration, that influence cell-fate decisions in vivo. Such complementary approaches would not replace the Boolean framework but rather build upon its qualitative insights to refine predictions and enhance their translational relevance. Analysis of public clinical datasets, such as TCGA or METABRIC, would be a valuable next step to assess the translational relevance of our findings. In addition, the regulatory network was constructed from literature-curated interactions and is therefore not specific to any single HCC cell line. While this provides a generic framework for identifying core regulatory logic, the known genetic heterogeneity of HCC may influence the behavior of this network in different cellular contexts. Future work should validate the predicted feedback loops in HCC cell lines with defined genetic backgrounds—such as HepG2, Huh7, or Hep3B—to assess the generalizability of our findings and to refine the model for patient-specific applications. Furthermore, the genetic heterogeneity of HCC represents a major challenge for interpreting and translating our findings. Our model provides a generic framework that should be refined and validated in patient-specific or subtype-specific contexts to account for this heterogeneity. We also emphasize that the proposed therapeutic strategies are computational predictions that require experimental and clinical validation.

Despite these limitations, we believe our study offers translational value. While the work is hypothesis-generating rather than clinically ready, we have identified a novel and experimentally testable double-negative feedback loop involving MYC, SNHG7, and miR-34a. The model further predicts that restoring miR-34a activity or targeting SNHG7 could shift the system toward cell death. Future studies could extend this framework to patient-specific data, enabling personalized network analyses. We therefore propose our model as a platform for hypothesis generation and experimental prioritisation, which we consider a meaningful translational contribution.

Second, the present network does not incorporate the full complexity of the HCC tumor microenvironment. Third, although the model was validated against independent experimental observations, direct experimental validation of the predicted MYC/SNHG7/miR-34a feedback loop remains necessary. Future studies integrating transcriptomic, proteomic, and functional perturbation data will be important for refining the model and evaluating its clinical relevance.

## 4. Methods

### 4.1. Construction of the MYC/SNHG7/miR-34a-5p/SIRT1/NLRP3 Regulatory Network in HCC

A mechanistic regulatory network describing the MYC/SNHG7/miR-34a-5p/SIRT1/NLRP3 axis in HCC was assembled through systematic literature curation. Experimentally supported molecular interactions were retrieved from published studies indexed in PubMed and further verified using curated interaction databases [66] and miRTargetlink [67]. Only experimentally validated activating and inhibitory interactions were incorporated into the final network. The resulting regulatory network was constructed from literature-curated molecular interactions and is therefore not derived from any single HCC cell line. This cell-line-independent framework is based on consensus molecular interactions and is designed to capture core regulatory logic that may be conserved across HCC contexts. The resulting regulatory map was implemented as a Boolean model using GINsim (version 3.1) [68]. The complete model file is provided in the Data Availability Section.

### 4.2. Boolean Modeling, Dynamic Simulations, and Perturbation Analysis

The regulatory network was translated into a Boolean dynamic framework in which nodes represent molecular regulators or phenotypic states, while directed edges denote activating or inhibitory interactions [68,69]. Each node was assigned a binary state (“0” for inactive and “1” for active), and node transitions were governed by logical operators derived from experimental evidence [60]. The Boolean rules used in the model are summarized in Table S1.

Network dynamics were analyzed using asynchronous updating schemes implemented in GINsim, allowing individual nodes to update independently and thereby approximating the stochastic nature of intracellular signaling dynamics. To estimate phenotypic distributions, Monte Carlo simulations were performed from randomly initialized conditions, and attractor probabilities were calculated according to the proportion of trajectories converging to each stable state [69].

Stable attractors were interpreted as biologically distinct phenotypic outcomes, including proliferation, drug resistance, senescence, pyroptosis, and apoptosis. Regulatory feedback loops were analyzed to identify network motifs associated with multistability. Feedback loops can be classified into two categories: positive feedback loops, which contain an even number of inhibitory interactions and reinforce signals to promote multistability, and negative feedback loops, which contain an odd number of inhibitory interactions and counteract signals to promote homeostasis [14]. Gain-of-function and loss-of-function perturbations were simulated by constraining selected nodes to constitutively active or inactive states, respectively, independent of upstream regulatory inputs [69].

## 5. Conclusions

In summary, our findings support a model in which the MYC/SNHG7/miR-34a regulatory circuit functions as a multistable control module governing the balance between drug resistance and cell death in hepatocellular carcinoma. By integrating established miR-34a-mediated senescence and apoptosis with a potential pyroptotic pathway, this study provides a systems-level framework for understanding how non-coding RNA networks shape cell-fate decisions and identifies miR-34a restoration as a potential strategy for overcoming therapy resistance in HCC.

## Figures and Tables

**Figure 1:**
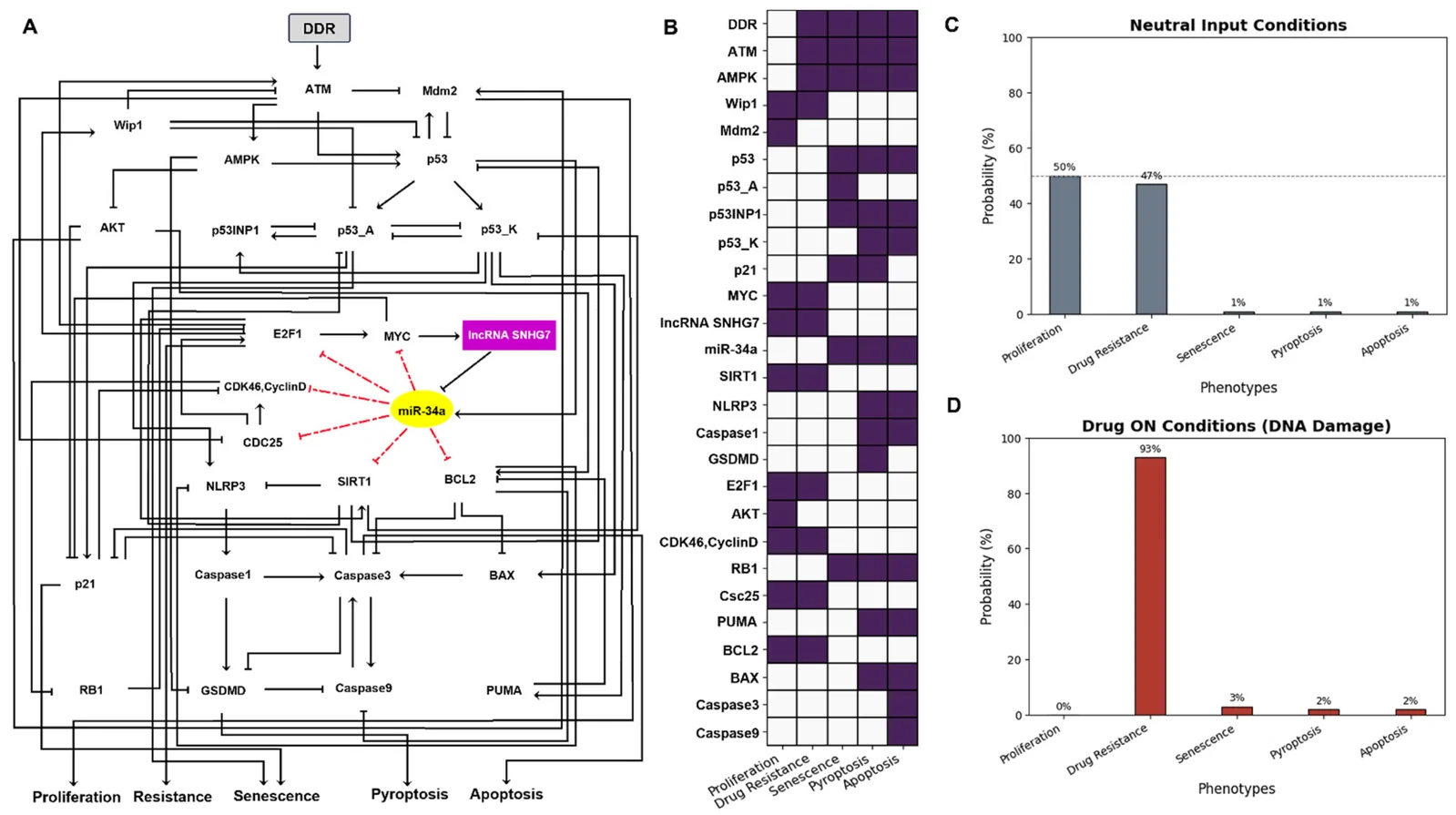
Boolean network model and wild-type attractor dynamics in HCC**.** (**A**) Regulatory network describing the SNHG7/miR-34a-5p/SIRT1 axis and its role in cell fate decisions under DNA damage response (DDR). The network integrates signaling proteins, transcription factors, one miRNA (miR-34a-5p; yellow oval node), and one lncRNA (SNHG7; pink rectangular node). Proteins and transcription factors are represented as white oval nodes. DDR is modeled as an external input node (gray rectangular node). Black arrows and hammerhead lines denote activating and inhibitory interactions, respectively. Dashed red lines represent miRNA-mediated repression of its targets. The phenotypic outputs include Proliferation, Drug Resistance, Senescence, Pyroptosis, and Apoptosis. (**B**) Wild-type attractor analysis. Each column represents a stable attractor, and each row a molecular node. Purple boxes indicate active state (1), white boxes indicate inactive state (0). Phenotypes are labeled at the bottom: Proliferation (column 1), Drug Resistance (column 2), Senescence (column 3), Pyroptosis (column 4), and Apoptosis (column 5). (**C**) Monte Carlo simulation probabilities (100,000 runs) with DDR input left free (neither fixed ON nor OFF). (**D**) Monte Carlo simulation probabilities (100,000 runs) with DDR fixed ON. Abbreviations: MYC, MYC proto-oncogene; p53, tumor protein p53; SNHG7, Small Nucleolar RNA Host Gene 7; miR-34a, microRNA-34a; SIRT1, Sirtuin 1; NLRP3, NLR family pyrin domain containing 3; GSDMD, Gasdermin D. For a complete list of all network components and their full names, see Table S1.

**Figure 2:**
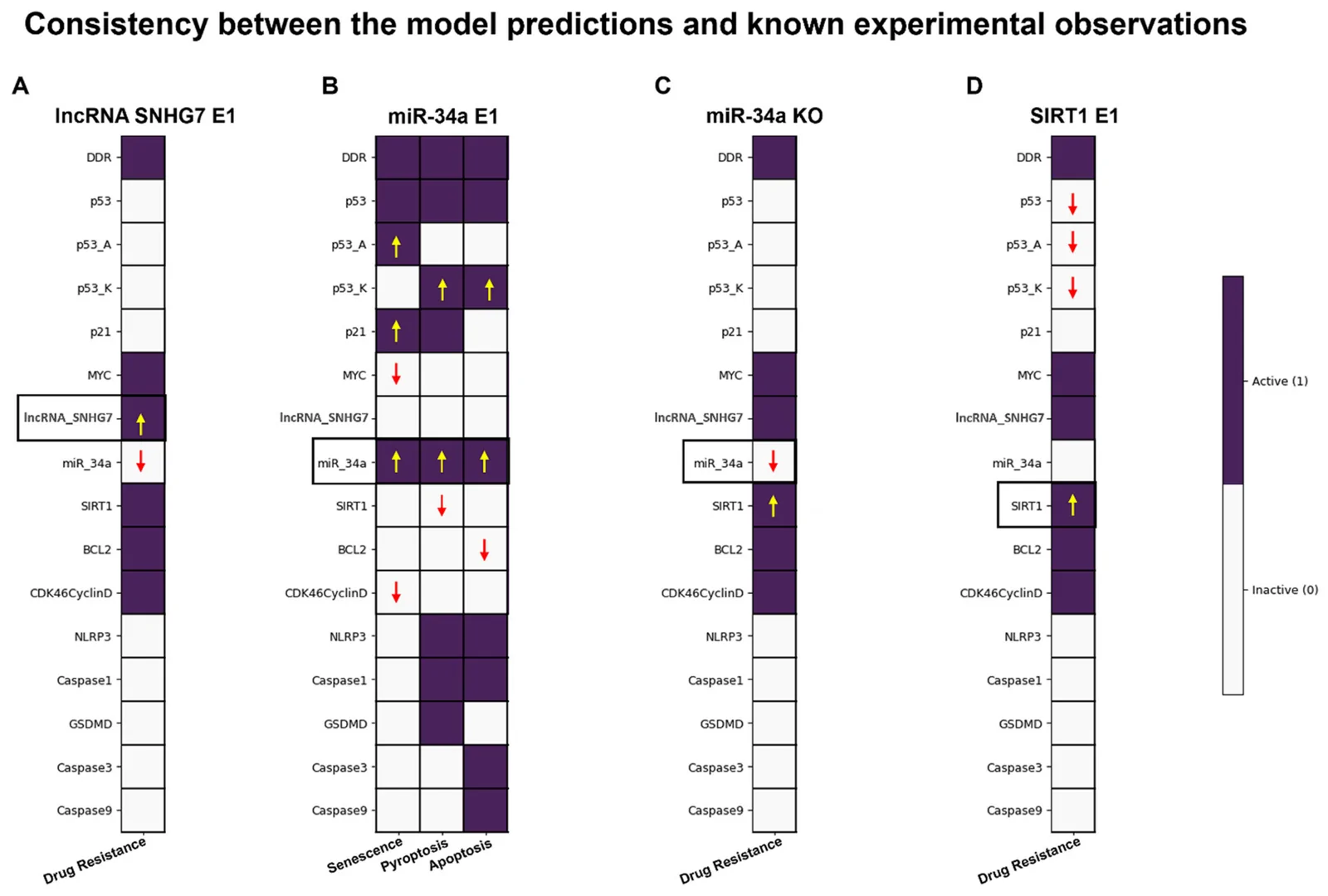
Model validation via gain-of-function (GoF) and loss-of-function (LoF) perturbation analysis. Model predictions under various mutant conditions are compared with established experimental phenotypes. Each column represents a stable state; each row shows the activity level of a molecular node. Purple boxes indicate the active state (1), and white boxes indicate the inactive state (0). Phenotypes are labeled at the bottom: Proliferation, Drug Resistance, Senescence, Pyroptosis, and Apoptosis. Yellow upward arrows indicate upregulation, and red downward arrows indicate downregulation of the corresponding molecules. Gain-of-function (E1) and loss-of-function (KO) perturbations were simulated by constitutively activating or knocking out the indicated nodes, respectively. (**A**) SNHG7 overexpression (GoF) stabilizes Drug Resistance, corresponding to Chen et al., 2020 [11]. (**B**) miR-34a-5p overexpression (GoF) inhibits Drug Resistance and induces Senescence, Pyroptosis, and Apoptosis, corresponding to Xu et al., 2015 [58], Chen et al., 2020 [11] and Yang et al., 2014 [59]. (**C**) miR-34a-5p knockdown (LoF) promotes Drug Resistance, corresponding to Chen et al., 2020 [11]. (**D**) SIRT1 overexpression (GoF) sustains Drug Resistance, corresponding to Chen et al., 2020 [11]. Experimental support for each prediction is detailed in Section 2.2.

**Figure 3:**
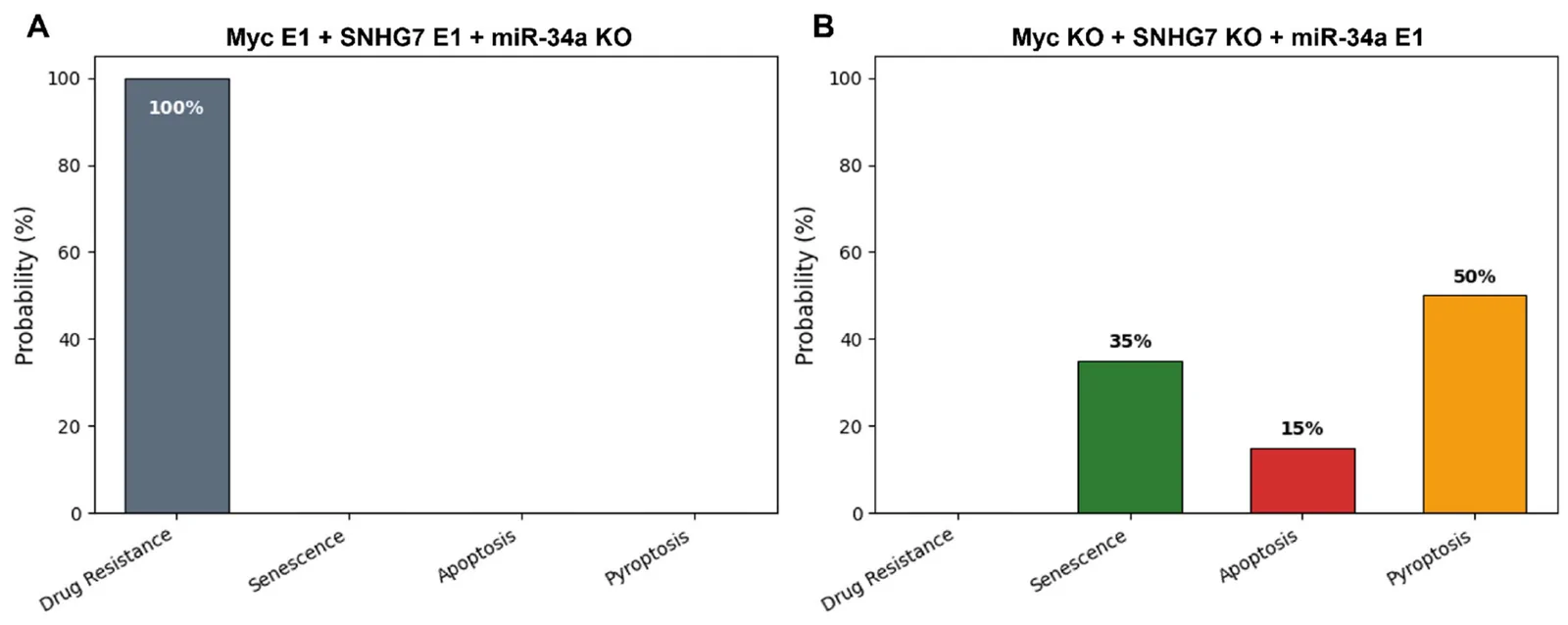
The MYC/SNHG7/miR-34a-5p loop functions as a multistable switch controlling drug resistance and cell death in HCC. (**A**) miR-34a-5p knockdown combined with MYC and SNHG7 overexpression locks the system into full Drug Resistance (100%), with complete suppression of Senescence, Pyroptosis, and Apoptosis. (**B**) miR-34a-5p overexpression combined with MYC and SNHG7 knockdown eliminates Drug Resistance and shifts the system toward cell death: Senescence (35%), Apoptosis (15%), and Pyroptosis (50%).

**Figure 4:**
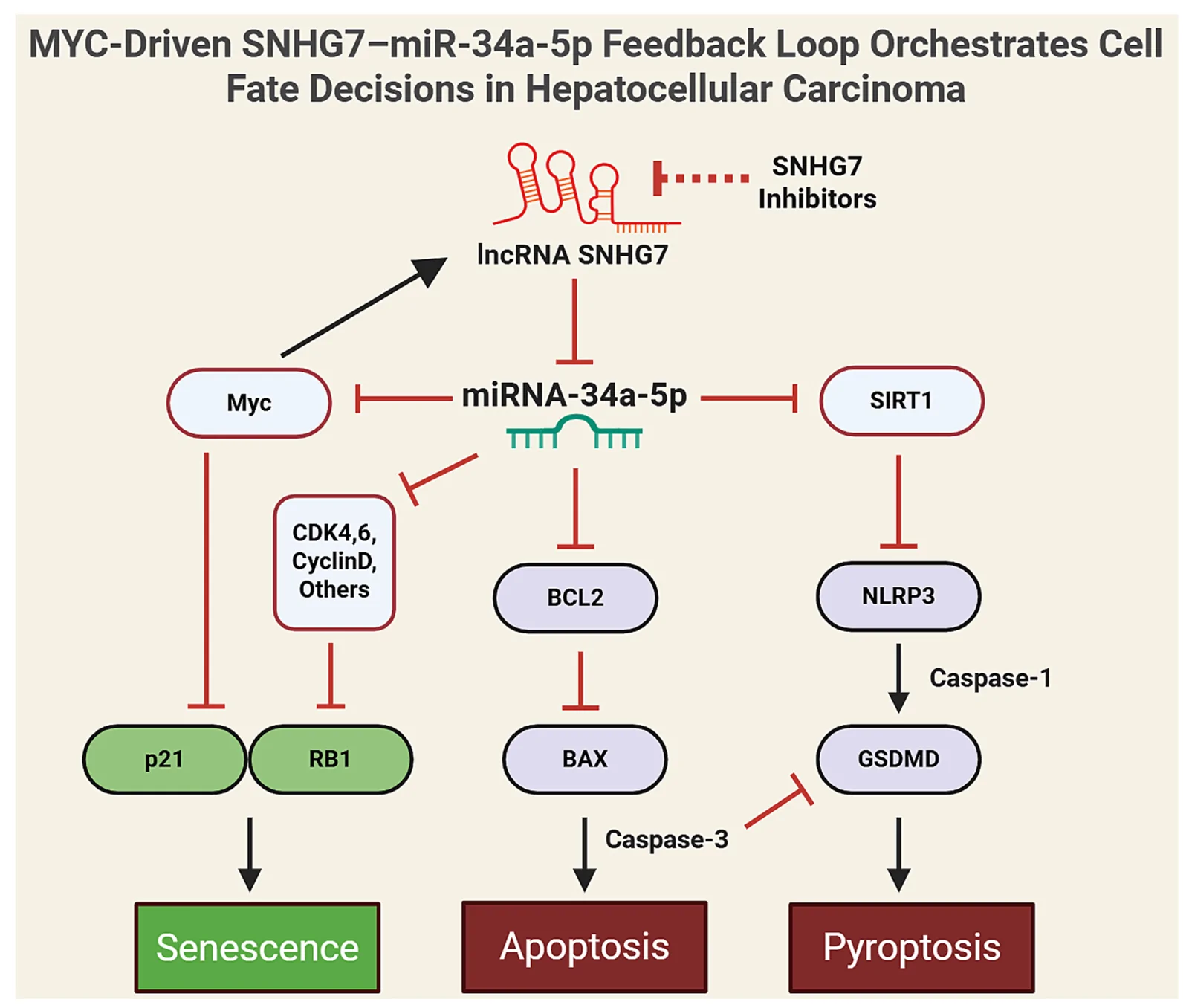
Proposed MYC–SNHG7–miR-34a-5p regulatory module governing cell-fate decisions in hepatocellular carcinoma. MYC drives the expression of SNHG7, which acts as a competing endogenous RNA that inhibits miR-34a-5p, thereby relieving repression of its downstream targets. In turn, miR-34a-5p directly represses MYC, forming a double-negative feedback loop. miR-34a-5p regulates multiple oncogenic and tumor-suppressive pathways, including suppression of BCL2-mediated survival signaling, thereby promoting the BAX/Caspase-3-mediated apoptotic pathway. In parallel, MYC influences cell-cycle regulators, including CDK4/6, Cyclin D, p21, and RB1, thereby controlling senescence outcomes. The SIRT1 axis modulates inflammasome activation through NLRP3 and downstream Caspase-1/GSDMD signaling, contributing to pyroptosis. Collectively, the proposed MYC–SNHG7–miR-34a-5p regulatory network governs the balance between senescence, apoptosis, and pyroptosis in HCC. Black arrows denote activating interactions, red hammerhead lines indicate inhibitory interactions, and dashed red hammerhead lines represent pharmacological inhibition (e.g., SNHG7 inhibition), which is predicted to restore miR-34a-5p activity and promote cell-fate switching. Abbreviations: MYC, MYC proto-oncogene; miR-34a-5p, microRNA-34a-5p; SNHG7, Small Nucleolar RNA Host Gene 7; SIRT1, Sirtuin 1; BCL2, B-cell lymphoma 2; BAX, BCL2 associated X protein; CDK4/6, Cyclin-dependent kinases 4/6; RB1, Retinoblastoma protein; NLRP3, NLR family pyrin domain containing 3; GSDMD, Gasdermin D. For a complete list of all network components and their full names, see Table S1.

**Table 1:** Regulatory interactions within the Myc/SNHG7/miR-34a double-negative feedback loop.

Circuit	Circuit Elements	Interaction Type	Biological Explanation	Ref.
MYC/SNHG7/miR-34a	MYC/SNHG7	Activation	MYC transcriptionally activates SNHG7 expression	[60]
	SNHG7/miR34a	Inhibition (ceRNA)	SNHG7 acts as a competing endogenous RNA, sponging miR-34a and reducing its availability	[10]
	miR-34a/MYC	Inhibition	miR-34a directly binds to the 3’UTR of MYC mRNA, repressing its translation	[58]

## Data Availability

The model code is available in a publicly accessible GitHub (version 3.6.5) repository, which can be accessed through the following link: https://github.com/gupatshan/MYC-SNHG7-miR34a-Boolean-Network-HCC (accessed on 30 August 2026).

## Supplementary Materials

The following supporting information can be downloaded at https://www.mdpi.com/article/10.3390/ijms27198441/s1.

## References

[1] Xie X., Wang Y., Wang Z., Zhang L., Li J., Li Y. (2025). Hepatocellular carcinoma drug resistance models. Cancer Cell Int..

[2] Zhao Y., Yang L., Wang P., Huo S., Yuan X., Jiang J. (2026). Molecular regulation and therapeutic targeting of programmed cell death in hepatocellular carcinoma. Pathol. Res. Pract..

[3] Bergsbaken T., Fink S.L., Cookson B.T. (2009). Pyroptosis: Host cell death and inflammation. Nat. Rev. Microbiol..

[4] Dai Z., Liu W.-C., Chen X.-Y., Wang X., Li J.-L., Zhang X. (2023). Gasdermin D-mediated pyroptosis: Mechanisms, diseases, and inhibitors. Front. Immunol..

[5] Lei K., Chen J., Deng Y., Peng Y., Zhai X., Ren X., Zhao J., Chen B., Jiang C. (2025). Cracking the code of cancer immunotherapy resistance: Emerging roles of pyroptosis and necroptosis. J. Exp. Clin. Cancer Res. CR.

[6] Aalijahan H., Ghorbian S. (2019). Long non-coding RNAs and cervical cancer. Exp. Mol. Pathol..

[7] Christoffersen N.R., Shalgi R., Frankel L.B., Leucci E., Lees M., Klausen M., Pilpel Y., Nielsen F.C., Oren M., Lund A.H. (2010). p53-independent upregulation of miR-34a during oncogene-induced senescence represses MYC. Cell Death Differ..

[8] Li L., Yuan L., Luo J., Gao J., Guo J., Xie X. (2013). MiR-34a inhibits proliferation and migration of breast cancer through down-regulation of Bcl-2 and SIRT1. Clin. Exp. Med..

[9] Dang Y., Luo D., Rong M., Chen G. (2013). Underexpression of miR-34a in Hepatocellular Carcinoma and Its Contribution towards Enhancement of Proliferating Inhibitory Effects of Agents Targeting c-MET. PLoS ONE.

[10] Pei L.-J., Sun P.-J., Ma K., Guo Y.-Y., Wang L.-Y., Liu F.-D. (2021). LncRNA-SNHG7 interferes with miR-34a to de-sensitize gastric cancer cells to cisplatin. Cancer Biomark..

[11] Chen Z., He M., Chen J., Li C., Zhang Q. (2020). Long non-coding RNA SNHG7 inhibits NLRP3-dependent pyroptosis by targeting the miR-34a/SIRT1 axis in liver cancer. Oncol. Lett..

[12] Barbuti R., Gori R., Milazzo P., Nasti L. (2020). A survey of gene regulatory networks modelling methods: From differential equations, to Boolean and qualitative bioinspired models. J. Membr. Comput..

[13] Schwab J.D., Kühlwein S.D., Ikonomi N., Kühl M., Kestler H.A. (2020). Concepts in Boolean network modeling: What do they all mean?. Comput. Struct. Biotechnol. J..

[14] Mombach J.C.M., Bugs C.A., Sinigaglia M., Gupta S., Silveira D.A. (2026). Systems biology of gene regulation: Qualitative simulation with Boolean networks. npj Syst. Biol. Appl..

[15] Thomas R., Thieffry D., Kaufman M. (1995). Dynamical behaviour of biological regulatory networks--I. Biological role of feedback loops and practical use of the concept of the loop-characteristic state. Bull. Math. Biol..

[16] Thieffry D. (2007). Dynamical roles of biological regulatory circuits. Brief. Bioinform..

[17] Gupta S., Hashimoto R.F. (2022). Dynamical Analysis of a Boolean Network Model of the Oncogene Role of lncRNA ANRIL and lncRNA UFC1 in Non-Small Cell Lung Cancer. Biomolecules.

[18] Gupta S., Panda P.K., Hashimoto R.F., Samal S.K., Mishra S., Verma S.K., Mishra Y.K., Ahuja R. (2022). Dynamical modeling of miR-34a, miR-449a, and miR-16 reveals numerous DDR signaling pathways regulating senescence, autophagy, and apoptosis in HeLa cells. Sci. Rep..

[19] Gupta S., Silveira D.A., Mombach J.C.M. (2020). Towards DNA-damage induced autophagy: A Boolean model of p53-induced cell fate mechanisms. DNA Repair.

[20] Gupta S., Silveira D.A., Mombach J.C.M., Hashimoto R.F. (2025). DNA Damage-Induced Ferroptosis: A Boolean Model Regulating p53 and Non-Coding RNAs in Drug Resistance. Proteomes.

[21] Gupta S., Silveira D.A., Dalmolin R.J.S., Mombach J.C.M., Hashimoto R.F. (2026). p53 Orchestrates the Immunogenic-Tolerogenic Pyroptosis Switch in Non-Small Cell Lung Cancer: A Systems Biology Approach. Comput. Struct. Biotechnol. J..

[22] Llovet J.M., Ricci S., Mazzaferro V., Hilgard P., Gane E., Blanc J.-F., de Oliveira A.C., Santoro A., Raoul J.-L., Forner A. (2008). Sorafenib in advanced hepatocellular carcinoma. N. Engl. J. Med..

[23] Kudo M., Finn R.S., Qin S., Han K.-H., Ikeda K., Piscaglia F., Baron A., Park J.-W., Han G., Jassem J. (2018). Lenvatinib versus sorafenib in first-line treatment of patients with unresectable hepatocellular carcinoma: A randomised phase 3 non-inferiority trial. Lancet.

[24] Bruix J., Qin S., Merle P., Granito A., Huang Y.-H., Bodoky G., Pracht M., Yokosuka O., Rosmorduc O., Breder V. (2017). Regorafenib for patients with hepatocellular carcinoma who progressed on sorafenib treatment (RESORCE): A randomised, double-blind, placebo-controlled, phase 3 trial. Lancet.

[25] Liu J., Yu Y., Xu B., Liang Q., Fang T., Zhou N., Sun G. (2024). NOTCH1 regulates the DNA damage response and sorafenib resistance by activating ATM in hepatocellular carcinoma. Am. J. Transl. Res..

[26] Zhang S., Guan M., Ren T., Li N., Ding Q., Sun D., Zhu H. (2025). Prexasertib exerts a synergistic effect on the antitumor activity of Lenvatinib through ALOX15-mediated ferroptosis in hepatocellular carcinoma. Int. Immunopharmacol..

[27] Bi W., Sun X., Yi Q., Jiang X., He H., Jiang L., Fan Z., Huang H., Wen W., Jiang X. (2025). PRMT5 attenuates regorafenib-induced DNA damage in hepatocellular carcinoma cells through symmetric dimethylation of RPL14. J. Gastrointest. Oncol..

[28] Li J., Yong T., Chen Y., Zeng T., Zhang K., Wang S., Zhang Y. (2025). Targeting PCNA/PARP1 axis inhibits the malignant progression of hepatocellular carcinoma. Front. Pharmacol..

[29] Wang H., Wang K., Zheng X., Dong D., Zhu X., Sui X., Teng H., Xing B., Wang W. (2026). Camrelizumab, Apatinib, and Radiotherapy in Locally Advanced, Unresectable Hepatocellular Carcinoma: A Phase II Study. Clin. Cancer Res..

[30] Finn R.S., Qin S., Ikeda M., Galle P.R., Ducreux M., Kim T.-Y., Kudo M., Breder V., Merle P., Kaseb A.O. (2020). Atezolizumab plus Bevacizumab in Unresectable Hepatocellular Carcinoma. N. Engl. J. Med..

[31] Wang Z., Inuzuka H., Zhong J., Fukushima H., Wan L., Liu P., Wei W. (2012). DNA damage-induced activation of ATM promotes β-TRCP-mediated Mdm2 ubiquitination and destruction. Oncotarget.

[32] Luo L., Huang W., Tao R., Hu N., Xiao Z.-X., Luo Z. (2013). ATM and LKB1 dependent activation of AMPK sensitizes cancer cells to etoposide-induced apoptosis. Cancer Lett..

[33] Cheng Q., Chen L., Li Z., Lane W.S., Chen J. (2009). ATM activates p53 by regulating MDM2 oligomerization and E3 processivity. EMBO J..

[34] Lee C.-W., Wong L.L.-Y., Tse E.Y.-T., Liu H.-F., Leong V.Y.-L., Lee J.M.-F., Hardie D.G., Ng I.O.-L., Ching Y.-P. (2012). AMPK promotes p53 acetylation via phosphorylation and inactivation of SIRT1 in liver cancer cells. Cancer Res..

[35] Tang Y., Zhao W., Chen Y., Zhao Y., Gu W. (2008). Acetylation Is Indispensable for p53 Activation. Cell.

[36] Zhang X.-P., Liu F., Wang W. (2011). Two-phase dynamics of p53 in the DNA damage response. Proc. Natl. Acad. Sci. USA.

[37] Song C., Lu P., Sun G., Yang L., Wang Z., Wang Z. (2017). miR-34a sensitizes lung cancer cells to cisplatin via p53/miR-34a/MYCN axis. Biochem. Biophys. Res. Commun..

[38] Park E.Y., Woo Y., Kim S.J., Kim D.H., Lee E.K., De U., Kim K.S., Lee J., Jung J.H., Ha K.-T. (2016). Anticancer Effects of a New SIRT Inhibitor, MHY2256, against Human Breast Cancer MCF-7 Cells via Regulation of MDM2-p53 Binding. Int. J. Biol. Sci..

[39] Nakano K., Vousden K.H. (2001). PUMA, a novel proapoptotic gene, is induced by p53. Mol. Cell.

[40] Krześniak M., Zajkowicz A., Gdowicz-Kłosok A., Głowala-Kosińska M., Łasut-Szyszka B., Rusin M. (2020). Synergistic activation of p53 by actinomycin D and nutlin-3a is associated with the upregulation of crucial regulators and effectors of innate immunity. Cell. Signal..

[41] Chen Z., Wang L., Yao D., Yang T., Cao W.-M., Dou J., Pang J.C., Guan S., Zhang H., Yu Y. (2016). Wip1 inhibitor GSK2830371 inhibits neuroblastoma growth by inducing Chk2/p53-mediated apoptosis. Sci. Rep..

[42] Cregan S.P., MacLaurin J.G., Craig C.G., Robertson G.S., Nicholson D.W., Park D.S., Slack R.S. (1999). Bax-dependent caspase-3 activation is a key determinant in p53-induced apoptosis in neurons. J. Neurosci. Off. J. Soc. Neurosci..

[43] Yamamura S., Saini S., Majid S., Hirata H., Ueno K., Deng G., Dahiya R. (2012). MicroRNA-34a modulates c-Myc transcriptional complexes to suppress malignancy in human prostate cancer cells. PLoS ONE.

[44] Duan K., Ge Y.-C., Zhang X.-P., Wu S.-Y., Feng J.-S., Chen S.-L., Zhang L.I., Yuan Z.-H., Fu C.-H. (2015). miR-34a inhibits cell proliferation in prostate cancer by downregulation of SIRT1 expression. Oncol. Lett..

[45] Han R., Chen X., Li Y., Zhang S., Li R., Lu L. (2019). MicroRNA-34a suppresses aggressiveness of hepatocellular carcinoma by modulating E2F1, E2F3, and Caspase-3. Cancer Manag. Res..

[46] Liu J., Yuan J.-F., Wang Y.-Z. (2022). METTL3-stabilized lncRNA SNHG7 accelerates glycolysis in prostate cancer via SRSF1/c-Myc axis. Exp. Cell Res..

[47] Deng Y., Zhao F., Zhang Z., Sun F., Wang M. (2018). Long Noncoding RNA SNHG7 Promotes the Tumor Growth and Epithelial-to-Mesenchymal Transition via Regulation of miR-34a Signals in Osteosarcoma. Cancer Biother. Radiopharm..

[48] Sun Z., Lu W., Lin N., Lin H., Zhang J., Ni T., Meng L., Zhang C., Guo H. (2020). Dihydromyricetin alleviates doxorubicin-induced cardiotoxicity by inhibiting NLRP3 inflammasome through activation of SIRT1. Biochem. Pharmacol..

[49] Xu Z., Wang H., Qin Z., Zhao F., Zhou L., Xu L., Jia R. (2021). NLRP3 inflammasome promoted the malignant progression of prostate cancer via the activation of caspase-1. Cell Death Discov..

[50] Zhang T., Li Y., Zhu R., Song P., Wei Y., Liang T., Xu G. (2019). Transcription Factor p53 Suppresses Tumor Growth by Prompting Pyroptosis in Non-Small-Cell Lung Cancer. Oxid. Med. Cell. Longev..

[51] Chou C.-C., Lee K.-H., Lai I.-L., Wang D., Mo X., Kulp S.K., Shapiro C.L., Chen C.-S. (2014). AMPK reverses the mesenchymal phenotype of cancer cells by targeting the Akt-MDM2-Foxo3a signaling axis. Cancer Res..

[52] Ogawara Y., Kishishita S., Obata T., Isazawa Y., Suzuki T., Tanaka K., Masuyama N., Gotoh Y. (2002). Akt enhances Mdm2-mediated ubiquitination and degradation of p53. J. Biol. Chem..

[53] Dalmases A., González I., Menendez S., Arpí O., Corominas J.M., Servitja S., Tusquets I., Chamizo C., Rincón R., Espinosa L. (2014). Deficiency in p53 is required for doxorubicin induced transcriptional activation of NF-κB target genes in human breast cancer. Oncotarget.

[54] Sun F., Fu H., Liu Q., Tie Y., Zhu J., Xing R., Sun Z., Zheng X. (2008). Downregulation of CCND1 and CDK6 by miR-34a induces cell cycle arrest. FEBS Lett..

[55] Lu Z., Bauzon F., Fu H., Cui J., Zhao H., Nakayama K., Nakayama K.I., Zhu L. (2014). Skp2 suppresses apoptosis in Rb1-deficient tumours by limiting E2F1 activity. Nat. Commun..

[56] Capparelli C., Chiavarina B., Whitaker-Menezes D., Pestell T.G., Pestell R.G., Hulit J., Andò S., Howell A., Martinez-Outschoorn U.E., Sotgia F. (2012). CDK inhibitors (p16/p19/p21) induce senescence and autophagy in cancer-associated fibroblasts, “fueling” tumor growth via paracrine interactions, without an increase in neo-angiogenesis. Cell Cycle.

[57] Zheng S.-Z., Sun P., Wang J.-P., Liu Y., Gong W., Liu J. (2019). MiR-34a overexpression enhances the inhibitory effect of doxorubicin on HepG2 cells. World J. Gastroenterol..

[58] Xu X., Chen W., Miao R., Zhou Y., Wang Z., Zhang L., Wan Y., Dong Y., Qu K., Liu C. (2015). miR-34a induces cellular senescence via modulation of telomerase activity in human hepatocellular carcinoma by targeting FoxM1/c-Myc pathway. Oncotarget.

[59] Yang F., Li Q., Gong Z., Zhou L., You N., Wang S., Li X., Li J., An J., Wang D. (2014). MicroRNA-34a targets Bcl-2 and sensitizes human hepatocellular carcinoma cells to sorafenib treatment. Technol. Cancer Res. Treat..

[60] Zhang L., Fu Y., Guo H. (2019). c-Myc-Induced Long Non-Coding RNA Small Nucleolar RNA Host Gene 7 Regulates Glycolysis in Breast Cancer. J. Breast Cancer.

[61] Gupta S. (2026). The p21 resilience network: A conceptual framework linking senescence to ferroptosis and cuproptosis resistance. npj Syst. Biol. Appl..

[62] Hong D.S., Kang Y.-K., Borad M., Sachdev J., Ejadi S., Lim H.Y., Brenner A.J., Park K., Lee J.-L., Kim T.-Y. (2020). Phase 1 study of MRX34, a liposomal miR-34a mimic, in patients with advanced solid tumours. Br. J. Cancer.

[63] Pranatharthiharan S., Patel M.D., Malshe V.C., Pujari V., Gorakshakar A., Madkaikar M., Ghosh K., Devarajan P.V. (2017). Asialoglycoprotein receptor targeted delivery of doxorubicin nanoparticles for hepatocellular carcinoma. Drug Deliv..

[64] Zhou X.-Q., Li Y.-P., Dang S.-S. (2024). Precision targeting in hepatocellular carcinoma: Exploring ligand-receptor mediated nanotherapy. World J. Hepatol..

[65] Xiao Z., Li C.H., Chan S.L., Xu F., Feng L., Wang Y., Jiang J.-D., Sung J.J.Y., Cheng C.H.K., Chen Y. (2014). A Small-Molecule Modulator of the Tumor-Suppressor miR34a Inhibits the Growth of Hepatocellular Carcinoma. Cancer Res..

[66] Oughtred R., Stark C., Breitkreutz B.-J., Rust J., Boucher L., Chang C., Kolas N., O’Donnell L., Leung G., McAdam R. (2019). The BioGRID interaction database: 2019 update. Nucleic Acids Res..

[67] Hamberg M., Backes C., Fehlmann T., Hart M., Meder B., Meese E., Keller A. (2016). MiRTargetLink--miRNAs, Genes and Interaction Networks. Int. J. Mol. Sci..

[68] Naldi A., Hernandez C., Abou-Jaoudé W., Monteiro P.T., Chaouiya C., Thieffry D. (2018). Logical Modeling and Analysis of Cellular Regulatory Networks with GINsim 3.0. Front. Physiol..

[69] Abou-Jaoudé W., Traynard P., Monteiro P.T., Saez-Rodriguez J., Helikar T., Thieffry D., Chaouiya C. (2016). Logical Modeling and Dynamical Analysis of Cellular Networks. Front. Genet..

