# Supplementary material for: MYC–SNHG7–miR-34a-5p Feedback Loop Orchestrates GSDMD-Dependent Pyroptosis in Hepatocellular Carcinoma
Source: Int J Mol Sci. 2026 Sep 22;27(19):8441. doi: 10.3390/ijms27198441 (PMC13665239; doi:10.3390/ijms27198441)
Supplement: Supplementary file 1 [file ijms-27-08441-s001.zip › Table S1.pdf]

Supplementary Table S1

Table S1. Boolean rules and regulatory interactions of the MYC–SNHG7–miR-34a-5p network in hepatocellular carcinoma. The table lists all 32 signaling components, one miRNA (miR-34a-5p), one lncRNA (SNHG7), and five phenotypic outputs (Proliferation, Drug Resistance, Senescence, Pyroptosis, and Apoptosis) included in the Boolean network model, along with their corresponding logical rules derived from experimentally validated interactions. For each target node, the table specifies: (i) the official name of each molecule, (ii) the target node identifier used in the model, (iii) interaction numbers corresponding to regulatory inputs, (iv) the nature of each interaction (activation or inhibition), (v) a brief mechanistic description of the interaction, and (vi) the primary literature reference supporting each interaction. Logical rules (AND, OR, NOT) define the activation conditions for each node based on its regulators. Rules were formulated to capture the qualitative dynamics of the network as described in the literature. References correspond to the numbered citations in the main text. A total of 76 regulatory interactions is included. This table provides complete transparency regarding the evidence base for all network edges and update rules used in model simulations.

**Official Name**	**Target node**	**Interaction number**	**Interaction**	**Description**	**Reference**
DNA Damage Response	DDR	1	ATM	DNA damage Response directly activates ATM	[1]
	Rule	DDR			ATM activate in the presence of DRR.
Ataxia-Telangiectasia Mutated	ATM	2	DDR	DDR activates ATM	[1]
		3	Wip1	Wip1 inhibits ATM	[2]
		4	E2F1	E2F1 induced ATM	[3]
	Rule	DDR AND (E2F1 OR NOT Wip1)			ATM activate in the presence of DDR, as long as E2F1 is present or Wip1 is absent.
E3 ubiquitin-protein ligase Mdm2	Mdm2	5	ATM	ATM inhibits Mdm2	[4]
		6	p53	p53 induced mdm2	[4]

		7	AKT	AKT induced Mdm2	[5]
	Rule	NOT ATM OR (p53 AND AKT)			MDM2 activate in the absence of ATM, or if both p53 and AKT are present
Wild-type p53-induced phosphatase 1	Wip1	8	E2F1	E2F1 induced E2F1	[6]
	Rule	E2F1			Wip1 activate in the presence of E2F1
AMP-activated protein kinase	AMPK	9	ATM	ATM induced AMPK	[7]
	Rule	ATM			AMPK activate in the presence of E2F1
Tumor suppressor p53	p53	10	ATM	ATM induced p53	[4]
		11	Wip1	Wip1 inhibits p53	[8]
		12	AMPK	AMPK induced p53	[9]
		13	Mdm2	Mdm2 inhibits p53	[10]
		14	SIRT1	SIRT1 inhibits p53	[11]
	Rule	ATM AND (NOT Wip1 OR AMPK) AND NOT Mdm2 OR NOT SIRT1			P53 activate in the absence of SIRT1, OR when ATM is present, Mdm2 is absent, and either AMPK is present or Wip1 is absent.
Protein Kinase B	AKT	15	AMPK	AMPK inhibits AKT	[12]

	Rule	NOT AMPK			AKT activate in the absence of AMPK
Tumor protein p53 inducible nuclear protein 1	p53INP1	16	p53_A	p53_A induced p53INP1	[13]
		17	p53_K	p53_K induced p53INP1	[13]
	Rule	p53_A OR p53_K			p53INP1 activate in the presence of p53 A or p53 K
Tumor supressor p53 protein (Ser 15 and Ser 20)	p53_A	18	p53_K	P53_k inhibits p53_a	[14]
		19	Wip1	Wip1 inhibits p53_a	[15]
		20	p53INPI	p53INP1 inhibits p53_a	[16]
		21	SIRT1	SIRT1 inhibits p53_A	[17]
		22	p53	P53 induced p53_A	[17]
	Rule	NOT p53_K AND (NOT Wip1 OR NOT p53INP1) AND NOT SIRT1 AND p53			p53_A activates if p53 is present, p53_K and SIRT1 are absent, and either Wip1 or p53INP1 is absent.
Tumor supressor p53 protein (Ser 46)	p53_K	23	p53_A	p53_A inhibits p53_K	[13]
		24	p53	P53 induced p53_K	[13]
		25	SIRT1	SIRT1 inhibits p53_K	[18]
	Rule	NOT p53_A AND p53 AND NOT SIRT1			p53_K activate in the absence of p53_A and SIRT1 or in the presence of p53

Cyclin-dependent kinase 4/6-Cyclin D complex	CDK46CyclinD	26	p21	P21 inhibits CDK46Cyclin D	[19]
		27	Cdc25	CDC25 induced CDK46Cyclin D	[20]
		28	miR_34a	miR_34a inhibits CDK46Cyclin D	[21]
	Rule	(NOT p21 AND Cdc25) OR NOT miR_34a			CDK46CyclinD activates in the absence of miR_34a; OR if Cdc25 is present and p21 is absent
Myc proto-oncogene	MYC	29	E2F1	E2F1 induced MYC	[22]
		30	miR_34a	miR_34a inhibits MYC	[23]
	Rule	E2F1 OR NOT miR_34a			MYC activate in the presence of E2F1 or in the absence of miR_34a
Long non-coding RNA small nucleolar RNA host gene 7	lncRNA_SNHG7	31	MYC	MYC induced lncRNA_SNHG7	[24]
	Rule	MYC			lncRNA_SNHG7 activate in the presence of MYC
Sirtuin 1	SIRT1	32	E2F1	E2F1 induced SIRT1	[25]
		33	miR_34a	miR_34a inhibits SIRT1	[26]
	Rule	E2F1 OR NOT miR_34a			SIRT1 activate in the presence of E2F1 and the abscess of miR34a
MicroRNA-34a-5p	miR_34a	34	p53	P53 induced miR_34a	[27]
		35	lncRNA_SNHG7	lncRNA_SNHG7 Inhibits miR_34a	[28]

	Rule	p53 AND NOT lncRNA_SNHG7			miR_34a activate in the presence of p53 and in the absence of lncRNA_SNHG7
cyclin-dependent kinase inhibitor 1	p21	36	p53_A	p53_A induced p21	[29]
		37	Caspase3	Caspase3 inhibits p21	[30]
		38	AKT	AKT inhibits p21	[31]
		39	MYC	MYC inhibits p21	[32]
	Rule	p53_A OR (NOT Caspase3 AND NOT AKT AND NOT MYC)			P21 activates if p53_A is present; OR if Caspase3, AKT, and MYC are all absent.
NLR family pyrin domain containing 3	NLRP3	40	p53_K	p53_K induced NLRP3	[33]
		41	BCL2	BCL2 inhibits NLRP3	[34]
		42	SIRT1	SIRT1 inhibits NLRP3	[35]
	Rule	p53_K AND NOT BCL2 AND NOT SIRT1			.NLRP3 activates in the presence of p53_K and in the absence of both BCL2 and SIRT1
Bcl-2-associated X protein	BAX	43	BCL2	BCL2 inhibits BAX	[36]
		44	p53_K	p53_K induced BAX	[37]
	Rule	NOT BCL2 AND p53_K			BAX activates in the presence of p53_K and in the absence of BCL2
B-cell lymphoma 2	BCL2	45	PUMA	PUMA inhibits BCL2	[38]

		46	miR_34a	miR_34a inhibits BCL2	[39]
		47	AKT	AKT induced BCL2	[38]
	Rule	NOT PUMA AND (NOT miR_34a OR AKT)			BCL2 activates in the absence of PUMA and miR_34a or the presence of AKT
E2F Transcription Factor 1	E2F1	48	RB1	RB1 directly inhibits E2F1	[40]
		49	Cdc25	Cdc25A activates E2F1 expression	[41]
		50	miR_34a	miR_34a inhibit E2F1	[42]
	Rule	(NOT RB1 AND Cdc25) OR NOT miR_34a			E2F1 activates in the presence of Cdc25 or in the absence of RB1 and miR_34a
Caspase1	Casp1	51	NLRP3	NLRP3 induce Casp1	[43]
	Rule	NLRP3			Casp1 activates in the presence fo NLRP3
Caspase3	Casp3	52	BAX	BAX enhances Caspase3 activity	[44]
		53	Caspase9	Caspase9 increase Caspase3 activation	[45]
		54	BCL2	BCL2 indirect inhibit Casp3	[46]
		55	Caspase1	Caspase1 induce Caspase3	[47]
		56	p21	p21 inhibits Caspase3	[48]

	Rule	(BAX AND Caspase9) OR (NOT BCL2 AND Caspase1 AND NOT p21)			Casp3 activates if both BAX and Caspase9 are present, or if Caspase1 is present while both BCL2 and p21 are absent
p53 upregulated modulator of apoptosis	PUMA	57	p53_K	p53_K induce PUMA	[49]
	Rule	p53_K			PUMA activates in the presence of p53_K
Cell Division Cycle 25	Cdc25	58	ATM	ATM directly inhibits Cdc25A	[50]
		59	miR_34a	miR_34a downregulate Cdc25	[51]
	Rule	NOT ATM OR NOT miR_34a			Cdc25 activate in the absence of ATM or miR_34a
Retinoblastoma protein	RB1	60	CDK46CyclinD	CDK46-CycD directly inhibits RB1	[52]
	Rule	NOT CDK46CyclinD			RB1 activate in the absence of CDK46CyclinD
Gasdermin D	GSDMD	61	Caspase1	Casp1 induced GSDMD	[53]
		62	Caspase3	Casp3 Inhibit GSDMD	[54]
		63	AMPK	AMPK inhibit GSDMD	[55]
	Rule	Caspase1 AND (NOT Caspase3 OR NOT AMPK)			GSDMD activates in the presence of Casp1 and the absence of Casp3 or AMPK
Caspase9	Casp9	64	BCL2	BCL2 promotes Caspase9 inhibition	[56]
		65	GSDMD	GSDMD inhibit Casp9	[57]

		66	Casp3	Caspase3 increase Caspase9 activation	[58]
	Rule	NOT BCL2 AND Caspase 3 AND NOT GSDMD			Caspase9 activate in the absence of BCL2 and GSDMD and in the presence of Caspase3
PROLIFERATION	Proliferation	67	MDM2	MDM2 induced Proliferation	[59]
	Rule	MDM2			MDM2 promotes oncogenic transformation and malignant growth in neoplastic tissues
RESISTANCE	Resistance	68	E2F1	E2F1 induced Resistance	[60]
	Rule	E2F1			Resistance occurs in the presence of E2F1
SENESCENCE	Senescence	69	p21	P21 induced senescence	[61]
		70	p53_A	p53_A induced Senescence	[62]
	Rule	p21 OR p53_A			Senescence occurs in the presence of p21 or p53_A
PYROPTOSIS	Pyroptosis	71	GSDMD	GSDMD induced Pyroptosis	[63]
	Rule	GSDMD			Pyroptosis occurs in the presence of GSDMD
APOPTOSIS	Apoptosis	72	Caspase3	Caspase3 induced Apoptosis	[64]
	Rule	Caspase3			Apoptosis occurs in the presence of Caaspase3

References:

[1] Z. Wang *et al.*, “DNA damage-induced activation of ATM promotes β-TRCP-mediated Mdm2 ubiquitination and destruction”, *Oncotarget*, v. 3, n. 9, p. 1026–1035, set. 2012, doi: 10.18632/oncotarget.640.

[2] S. Yin *et al.*, “Wip1 suppresses ovarian cancer metastasis through the ATM/AKT/Snail mediated signaling”, *Oncotarget*, v. 7, n. 20, p. 29359–29370, abr. 2016, doi: 10.18632/oncotarget.8833.

[3] J. T. Powers, S. Hong, C. N. Mayhew, P. M. Rogers, E. S. Knudsen, e D. G. Johnson, “E2F1 Uses the ATM Signaling Pathway to Induce p53 and Chk2 Phosphorylation and Apoptosis”, *Mol. Cancer Res.*, v. 2, n. 4, p. 203–214, abr. 2004, doi: 10.1158/1541-7786.203.2.4.

[4] Q. Cheng, L. Chen, Z. Li, W. S. Lane, e J. Chen, “ATM activates p53 by regulating MDM2 oligomerization and E3 processivity”, *EMBO J.*, v. 28, n. 24, p. 3857–3867, dez. 2009, doi: 10.1038/emboj.2009.294.

[5] Y. Ogawara *et al.*, “Akt Enhances Mdm2-mediated Ubiquitination and Degradation of p53 *”, *J. Biol. Chem.*, v. 277, n. 24, p. 21843–21850, jun. 2002, doi: 10.1074/jbc.M109745200.

[6] T. Hershko, K. Korotayev, S. Polager, e D. Ginsberg, “E2F1 Modulates p38 MAPK Phosphorylation via Transcriptional Regulation of ASK1 and Wip1 *”, *J. Biol. Chem.*, v. 281, n. 42, p. 31309–31316, out. 2006, doi: 10.1016/S0021-9258(19)84043-7.

[7] L. Luo, W. Huang, R. Tao, N. Hu, Z.-X. Xiao, e Z. Luo, “ATM and LKB1 dependent activation of AMPK sensitizes cancer cells to etoposide-induced apoptosis”, *Cancer Lett.*, v. 328, n. 1, p. 114–119, jan. 2013, doi: 10.1016/j.canlet.2012.08.034.

[8] Z. Chen *et al.*, “Wip1 inhibitor GSK2830371 inhibits neuroblastoma growth by inducing Chk2/p53-mediated apoptosis”, *Sci. Rep.*, v. 6, n. 1, p. 38011, dez. 2016, doi: 10.1038/srep38011.

[9] C.-W. Lee *et al.*, “AMPK Promotes p53 Acetylation via Phosphorylation and Inactivation of SIRT1 in Liver Cancer Cells”, *Cancer Res.*, v. 72, n. 17, p. 4394–4404, ago. 2012, doi: 10.1158/0008-5472.CAN-12-0429.

[10] A. Dalmases *et al.*, “Deficiency in p53 is required for doxorubicin induced transcriptional activation of NF-κB target genes in human breast cancer”, *Oncotarget*, v. 5, n. 1, p. 196–210, nov. 2013, doi: 10.18632/oncotarget.1556.

[11] E.-J. Kim, J.-H. Kho, M.-R. Kang, e S.-J. Um, “Active Regulator of SIRT1 Cooperates with SIRT1 and Facilitates Suppression of p53 Activity”, *Mol. Cell*, v. 28, n. 2, p. 277–290, out. 2007, doi: 10.1016/j.molcel.2007.08.030.

[12] C.-C. Chou *et al.*, “AMPK Reverses the Mesenchymal Phenotype of Cancer Cells by Targeting the Akt–MDM2–Foxo3a Signaling Axis”, *Cancer Res.*, v. 74, n. 17, p. 4783–4795, set. 2014, doi: 10.1158/0008-5472.CAN-14-0135.

[13] X.-P. Zhang, F. Liu, e W. Wang, “Two-phase dynamics of p53 in the DNA damage response”, *Proc. Natl. Acad. Sci.*, v. 108, n. 22, p. 8990–8995, maio 2011, doi: 10.1073/pnas.1100600108.

[14] Q. Li *et al.*, “PP2Cδ inhibits p300-mediated p53 acetylation via ATM/BRCA1 pathway to impede DNA damage response in breast cancer”, *Sci. Adv.*, v. 5, n. 10, p. eaaw8417, out. 2019, doi: 10.1126/sciadv.aaw8417.

[15] S. Pechackova, K. Burdova, J. Benada, P. Kleiblova, G. Jenikova, e L. Macurek, “Inhibition of WIP1 phosphatase sensitizes breast cancer cells to genotoxic

stress and to MDM2 antagonist nutlin3", *Oncotarget*, v. 7, n. 12, p. 14458–14475, fev. 2016, doi: 10.18632/oncotarget.7363.
[16] J. Shahbazi *et al.*, "Histone deacetylase 2 and N-Myc reduce p53 protein phosphorylation at serine 46 by repressing gene transcription of tumor protein 53-induced nuclear protein 1", *Oncotarget*, v. 5, n. 12, p. 4257–4268, maio 2014, doi: 10.18632/oncotarget.1991.
[17] E. Y. Park *et al.*, "Anticancer Effects of a New SIRT Inhibitor, MHY2256, against Human Breast Cancer MCF-7 Cells via Regulation of MDM2-p53 Binding", *Int. J. Biol. Sci.*, v. 12, n. 12, p. 1555–1567, dez. 2016, doi: 10.7150/ijbs.13833.
[18] Y. W. Yi, H. J. Kang, H. J. Kim, Y. Kong, M. M. Brown, e I. Bae, "Targeting Mutant p53 by a SIRT1 Activator YK3237 Inhibits the Proliferation of TripleNegative Breast Cancer Cells", *Oncotarget*, v. 4, n. 7, p. 984–994, jul. 2013, doi: 10.18632/oncotarget.1070.
[19] C. Capparelli *et al.*, "CDK inhibitors (p16/p19/p21) induce senescence and autophagy in cancer-associated fibroblasts, 'fueling' tumor growth via paracrine interactions, without an increase in neo-angiogenesis", *Cell Cycle*, v. 11, n. 19, p. 3599–3610, out. 2012, doi: 10.4161/cc.21884.
[20] J. C. Liu *et al.*, "Identification of CDC25 as a Common Therapeutic Target for Triple-Negative Breast Cancer", *Cell Rep.*, v. 23, n. 1, p. 112–126, abr. 2018, doi: 10.1016/j.celrep.2018.03.039.
[21] F. Sun *et al.*, "Downregulation of CCND1 and CDK6 by miR-34a induces cell cycle arrest", *FEBS Lett.*, v. 582, n. 10, p. 1564–1568, abr. 2008, doi: 10.1016/j.febslet.2008.03.057.
[22] S. Ladu, D. F. Calvisi, E. A. Conner, M. Farina, V. M. Factor, e S. S. Thorgeirsson, "E2F1 Inhibits c-Myc-Driven Apoptosis via PIK3CA/Akt/mTOR and COX-2 in a Mouse Model of Human Liver Cancer", *Gastroenterology*, v. 135, n. 4, p. 1322–1332, out. 2008, doi: 10.1053/j.gastro.2008.07.012.
[23] S. Yamamura *et al.*, "MicroRNA-34a Modulates c-Myc Transcriptional Complexes to Suppress Malignancy in Human Prostate Cancer Cells", *PLOS ONE*, v. 7, n. 1, p. e29722, jan. 2012, doi: 10.1371/journal.pone.0029722.
[24] J. Liu, J.-F. Yuan, e Y.-Z. Wang, "METTL3-stabilized lncRNA SNHG7 accelerates glycolysis in prostate cancer via SRSF1/c-Myc axis", *Exp. Cell Res.*, v. 416, n. 1, p. 113149, jul. 2022, doi: 10.1016/j.yexcr.2022.113149.
[25] C. Wang *et al.*, "Interactions between E2F1 and SirT1 regulate apoptotic response to DNA damage", *Nat. Cell Biol.*, v. 8, n. 9, p. 1025–1031, set. 2006, doi: 10.1038/ncb1468.
[26] K. Duan *et al.*, "miR-34a inhibits cell proliferation in prostate cancer by downregulation of SIRT1 expression", *Oncol. Lett.*, v. 10, n. 5, p. 3223–3227, nov. 2015, doi: 10.3892/ol.2015.3645.
[27] C. Song, P. Lu, G. Sun, L. Yang, Z. Wang, e Z. Wang, "miR-34a sensitizes lung cancer cells to cisplatin via p53/miR-34a/MYCN axis", *Biochem. Biophys. Res. Commun.*, v. 482, n. 1, p. 22–27, jan. 2017, doi: 10.1016/j.bbrc.2016.11.037.
[28] Y. Deng, F. Zhao, Z. Zhang, F. Sun, e M. Wang, "Long Noncoding RNA SNHG7 Promotes the Tumor Growth and Epithelial-to-Mesenchymal Transition via Regulation of miR-34a Signals in Osteosarcoma", *Cancer Biother. Radiopharm.*, v. 33, n. 9, p. 365–372, nov. 2018, doi: 10.1089/cbr.2018.2503.
[29] Y. Tang, W. Zhao, Y. Chen, Y. Zhao, e W. Gu, "Acetylation Is Indispensable for p53 Activation", *Cell*, v. 133, n. 4, p. 612–626, maio 2008, doi: 10.1016/j.cell.2008.03.025.

[30] J. A. Park, K.-W. Kim, S. I. Kim, e S. K. Lee, “Caspase 3 specifically cleaves p21WAF1/CIP1 in the earlier stage of apoptosis in SK-HEP-1 human hepatoma cells”, *Eur. J. Biochem.*, v. 257, n. 1, p. 242–248, 1998, doi: 10.1046/j.1432-1327.1998.2570242.x.

[31] Y.-L. Chang *et al.*, “MicroRNA-7 inhibits the stemness of prostate cancer stem-like cells and tumorigenesis by repressing KLF4/PI3K/Akt/p21 pathway”, *Oncotarget*, v. 6, n. 27, p. 24017–24031, jun. 2015, doi: 10.18632/oncotarget.4447.

[32] J. Seoane, H.-V. Le, e J. Massagué, “Myc suppression of the p21Cip1 Cdk inhibitor influences the outcome of the p53 response to DNA damage”, *Nature*, v. 419, n. 6908, p. 729–734, out. 2002, doi: 10.1038/nature01119.

[33] M. Krześniak, A. Zajkowicz, A. Gdowicz-Kłosok, M. Głowala-Kosińska, B. Łasut-Szyszka, e M. Rusin, “Synergistic activation of p53 by actinomycin D and nutlin-3a is associated with the upregulation of crucial regulators and effectors of innate immunity”, *Cell. Signal.*, v. 69, p. 109552, maio 2020, doi: 10.1016/j.cellsig.2020.109552.

[34] F. Luo *et al.*, “The BCL-2 inhibitor APG-2575 resets tumor-associated macrophages toward the M1 phenotype, promoting a favorable response to anti-PD-1 therapy via NLRP3 activation”, *Cell. Mol. Immunol.*, v. 21, n. 1, p. 60–79, jan. 2024, doi: 10.1038/s41423-023-01112-y.

[35] Z. Sun *et al.*, “Dihydromyricetin alleviates doxorubicin-induced cardiotoxicity by inhibiting NLRP3 inflammasome through activation of SIRT1”, *Biochem. Pharmacol.*, v. 175, p. 113888, maio 2020, doi: 10.1016/j.bcp.2020.113888.

[36] L. F. Gibson, J. Fortney, G. Magro, S. G. Ericson, J. P. Lynch, e K. S. Landreth, “Regulation of BAX and BCL-2 expression in breast cancer cells by chemotherapy”, *Breast Cancer Res. Treat.*, v. 55, n. 2, p. 107–117, maio 1999, doi: 10.1023/A:1006175811676.

[37] S. P. Cregan *et al.*, “Bax-Dependent Caspase-3 Activation Is a Key Determinant in p53-Induced Apoptosis in Neurons”, *J. Neurosci.*, v. 19, n. 18, p. 7860–7869, set. 1999, doi: 10.1523/JNEUROSCI.19-18-07860.1999.

[38] M. A. Khan *et al.*, “Phytochemicals from Ajwa dates pulp extract induce apoptosis in human triple-negative breast cancer by inhibiting AKT/mTOR pathway and modulating Bcl-2 family proteins”, *Sci. Rep.*, v. 11, n. 1, p. 10322, maio 2021, doi: 10.1038/s41598-021-89420-z.

[39] L. Li, L. Yuan, J. Luo, J. Gao, J. Guo, e X. Xie, “MiR-34a inhibits proliferation and migration of breast cancer through down-regulation of Bcl-2 and SIRT1”, *Clin. Exp. Med.*, v. 13, n. 2, p. 109–117, maio 2013, doi: 10.1007/s10238-012-0186-5.

[40] Z. Lu *et al.*, “Skp2 suppresses apoptosis in Rb1-deficient tumours by limiting E2F1 activity”, *Nat. Commun.*, v. 5, n. 1, p. 3463, mar. 2014, doi: 10.1038/ncomms4463.

[41] E. Vigo *et al.*, “CDC25A Phosphatase Is a Target of E2F and Is Required for Efficient E2F-Induced S Phase”, *Mol. Cell. Biol.*, v. 19, n. 9, p. 6379–6395, set. 1999, doi: 10.1128/MCB.19.9.6379.

[42] R. Han, X. Chen, Y. Li, S. Zhang, R. Li, e L. Lu, “MicroRNA-34a suppresses aggressiveness of hepatocellular carcinoma by modulating E2F1, E2F3, and Caspase-3”, *Cancer Manag. Res.*, v. 11, p. 2963–2976, abr. 2019, doi: 10.2147/CMAR.S202664.

[43] Z. Xu *et al.*, “NLRP3 inflammasome promoted the malignant progression of prostate cancer via the activation of caspase-1”, *Cell Death Discov.*, v. 7, n. 1, p. 399, dez. 2021, doi: 10.1038/s41420-021-00766-9.
[44] S. P. Cregan *et al.*, “Bax-Dependent Caspase-3 Activation Is a Key Determinant in p53-Induced Apoptosis in Neurons”, *J. Neurosci.*, v. 19, n. 18, p. 7860–7869, set. 1999, doi: 10.1523/JNEUROSCI.19-18-07860.1999.
[45] H. Liu, Y. Zhou, e L. Tang, “Caffeine induces sustained apoptosis of human gastric cancer cells by activating the caspase-9/caspase-3 signalling pathway”, *Mol. Med. Rep.*, v. 16, n. 3, p. 2445–2454, set. 2017, doi: 10.3892/mmr.2017.6894.
[46] “Targeting Apoptosis in Acute Myeloid Leukemia: Current Status and Future Directions of BCL-2 Inhibition with Venetoclax and Beyond - ProQuest”. Acesso em: 5 de junho de 2026. [Online]. Disponível em: https://www.proquest.com/openview/9d3b91e25a26e5ae068e0d2306c32008/1?pq-origsite=gscholar&cbl=1486337
[47] R. N. Winter, A. Kramer, A. Borkowski, e N. Kyprianou, “Loss of Caspase-1 and Caspase-3 Protein Expression in Human Prostate Cancer”, *Cancer Res.*, v. 61, n. 3, p. 1227–1232, fev. 2001.
[48] Y. Zhang, N. Fujita, e T. Tsuruo, “Caspase-mediated cleavage of p21Waf1/Cip1 converts cancer cells from growth arrest to undergoing apoptosis”, *Oncogene*, v. 18, n. 5, p. 1131–1138, fev. 1999, doi: 10.1038/sj.onc.1202426.
[49] K. Nakano e K. H. Vousden, “PUMA, a novel proapoptotic gene, is induced by p53”, *Mol. Cell*, v. 7, n. 3, p. 683–694, mar. 2001, doi: 10.1016/s1097-2765(01)00214-3.
[50] C. Agarwal, A. Tyagi, e R. Agarwal, “Gallic acid causes inactivating phosphorylation of cdc25A/cdc25C-cdc2 via ATM-Chk2 activation, leading to cell cycle arrest, and induces apoptosis in human prostate carcinoma DU145 cells”, *Mol. Cancer Ther.*, v. 5, n. 12, p. 3294–3302, dez. 2006, doi: 10.1158/1535-7163.MCT-06-0483.
[51] T. Jiang e H. Cheng, “miR-34a-5p blocks cervical cancer growth and migration by downregulating CDC25A”, *J. BUON Off. J. Balk. Union Oncol.*, v. 26, n. 5, p. 1768–1774, 2021.
[52] A. Tyagi, C. Agarwal, e R. Agarwal, “Inhibition of Retinoblastoma Protein (Rb) Phosphorylation at Serine Sites and an Increase in Rb-E2F Complex Formation by Silibinin in Androgen-dependent Human Prostate Carcinoma LNCaP Cells: Role in Prostate Cancer Prevention”, *Mol. Cancer Ther.*, v. 1, n. 7, p. 525–532, maio 2002.
[53] H. Yan *et al.*, “Cisplatin Induces Pyroptosis via Activation of MEG3/NLRP3/caspase-1/GSDMD Pathway in Triple-Negative Breast Cancer”, *Int. J. Biol. Sci.*, v. 17, n. 10, p. 2606–2621, jun. 2021, doi: 10.7150/ijbs.60292.
[54] Z. Zhang, H. Zhang, D. Li, X. Zhou, Q. Qin, e Q. Zhang, “Caspase-3-mediated GSDME induced Pyroptosis in breast cancer cells through the ROS/JNK signalling pathway”, *J. Cell. Mol. Med.*, v. 25, n. 17, p. 8159–8168, 2021, doi: 10.1111/jcmm.16574.
[55] X. Chu *et al.*, “Gasdermin D-mediated pyroptosis is regulated by AMPK-mediated phosphorylation in tumor cells”, *Cell Death Dis.*, v. 14, n. 7, p. 469, jul. 2023, doi: 10.1038/s41419-023-06013-6.
[56] H. Tamaki *et al.*, “Bcl-2 family inhibition sensitizes human prostate cancer cells to docetaxel and promotes unexpected apoptosis under caspase-9 inhibition”, *Oncotarget*, v. 5, n. 22, p. 11399–11412, out. 2014, doi: 10.18632/oncotarget.2550.

[57] J. Shi *et al.*, “Cleavage of GSDMD by inflammatory caspases determines pyroptotic cell death”, *Nature*, v. 526, n. 7575, p. 660–665, out. 2015, doi: 10.1038/nature15514.

[58] C. Blanc *et al.*, “Caspase-3 Is Essential for Procaspase-9 Processing and Cisplatin-induced Apoptosis of MCF-7 Breast Cancer Cells”, *Cancer Res.*, v. 60, n. 16, p. 4386–4390, ago. 2000.

[59] G. Teoh *et al.*, “MDM2 Protein Overexpression Promotes Proliferation and Survival of Multiple Myeloma Cells”, *Blood*, v. 90, n. 5, p. 1982–1992, set. 1997, doi: 10.1182/blood.V90.5.1982.

[60] M. T. Rosenfeldt *et al.*, “E2F1 drives chemotherapeutic drug resistance via ABCG2”, *Oncogene*, v. 33, n. 32, p. 4164–4172, ago. 2014, doi: 10.1038/onc.2013.470.

[61] Z. Han *et al.*, “Role of p21 in Apoptosis and Senescence of Human Colon Cancer Cells Treated with Camptothecin*”, *J. Biol. Chem.*, v. 277, n. 19, p. 17154–17160, maio 2002, doi: 10.1074/jbc.M112401200.

[62] W. Xue *et al.*, “Senescence and tumour clearance is triggered by p53 restoration in murine liver carcinomas”, *Nature*, v. 445, n. 7128, p. 656–660, fev. 2007, doi: 10.1038/nature05529.

[63] Z. Zhang, H. Zhang, D. Li, X. Zhou, Q. Qin, e Q. Zhang, “Caspase-3-mediated GSDME induced Pyroptosis in breast cancer cells through the ROS/JNK signalling pathway”, *J. Cell. Mol. Med.*, v. 25, n. 17, p. 8159–8168, 2021, doi: 10.1111/jcmm.16574.

[64] N. O’Donovan *et al.*, “Caspase 3 in Breast Cancer”, *Clin. Cancer Res.*, v. 9, n. 2, p. 738–742, fev. 2003.
