# Supplementary material for: MYC–SNHG7–miR-34a-5p Feedback Loop Orchestrates GSDMD-Dependent Pyroptosis in Hepatocellular Carcinoma
Source: Int J Mol Sci. 2026 Sep 22;27(19):8441. doi: 10.3390/ijms27198441 (PMC13665239; doi:10.3390/ijms27198441)
Supplement: Supplementary file 1 [file ijms-27-08441-s001.zip › Table S2.pdf]

Supplementary Table S2

Table S2: Boolean network functional circuits and experimental validation. Predicted positive feedback circuits are labeled as "predicted" where no direct experimental evidence was found to support their existence.

**Positve Feedback Loops**	**References**
p21/Caspase3	*[1]*
Caspase3/Caspase9	*[2]*
Myc/SNHG7/miR-34a	Predicted
p53_A/p53_K	*[3]*
miR-34a/SIRT1/p53	*[4]*
**Negative Feedback Loops**	**References**
p53/MDM2	*[5]*
p53_A/p53_A	*[3]*
E2F1/ATM/Cdc25	*[6]*

References:

1 Abbas T & Dutta A (2009) p21 in cancer: intricate networks and multiple activities. *Nat Rev Cancer* **9**, 400–414.

2 Fujita E, Egashira J, Urase K, Kuida K & Momoi T (2001) Caspase-9 processing by caspase-3 via a feedback amplification loop in vivo. *Cell Death Differ* **8**, 335–344.

3 Zhang X-P, Liu F & Wang W (2011) Two-phase dynamics of p53 in the DNA damage response. *Proc Natl Acad Sci U S A* **108**, 8990–8995.

4 Yamakuchi M & Lowenstein CJ (2009) MiR-34, SIRT1 and p53: the feedback loop. *Cell Cycle* **8**, 712–715.

5 Bar-Or RL, Maya R, Segel LA, Alon U, Levine AJ & Oren M (2000) Generation of oscillations by the p53-Mdm2 feedback loop: A theoretical and experimental study. *PNAS* **97**, 11250–11255.

6 Vigo E, Müller H, Prosperini E, Hateboer G, Cartwright P, Moroni MC & Helin K (1999) CDC25A Phosphatase Is a Target of E2F and Is Required for Efficient E2F-Induced S Phase. *Molecular and Cellular Biology* **19**, 6379–6395.
